# AGE-DEPENDENT CHANGES IN INTERVERTEBRAL DISC CELL MITOCHONDRIA AND BIOENERGETICS

**DOI:** 10.22203/eCM.v036a13

**Published:** 2018-10-18

**Authors:** R. Hartman, P. Patil, R. Tisherman, C. St. Croix, L.J. Niedernhofer, P.D. Robbins, F. Ambrosio, B. Van Houten, G. Sowa, N. Vo

**Affiliations:** 1 Department of Physical Medicine and Rehabilitation, University of Pittsburgh, 3471 5th Avenue, Pittsburgh, PA 15213, USA; 2 University of Pittsburgh Medical Centre Enterprises, Pittsburgh, PA 15213, USA; 3 Department of Orthopaedic Surgery, University of Pittsburgh, 200 Lothrop Street, E1641 Biomedical Science Tower, Pittsburgh, PA 15261, USA; 4 Centre for Biologic Imaging, University of Pittsburgh, BST S224, 3500 Terrace St, Pittsburgh, PA 15261, USA; 5 Institute on the Biology of Aging and Metabolism, Department of Biochemistry, Molecular Biology and Biophysics, University of Minnesota, 312 Church Street, Minneapolis, MN, USA; 6 Department of Bioengineering, University of Pittsburgh, 3700 O’Hara Street, Pittsburgh, PA 15213, USA; 7 Department of Pharmacology and Chemical Biology, University of Pittsburgh, 2.6e Hillman Cancer Centre, 5117 Centre Avenue, Pittsburgh, PA 15213, USA

**Keywords:** Mitochondria, bioenergetics, ageing, disc, senescence

## Abstract

Robust cellular bioenergetics is vital in the energy-demanding process of maintaining matrix homeostasis in the intervertebral disc. Age-related decline in disc cellular bioenergetics is hypothesised to contribute to the matrix homeostatic perturbation observed in intervertebral disc degeneration. The present study aimed to measure how ageing impacted disc cell mitochondria and bioenergetics. Age-related changes measured included matrix content and cellularity in disc tissue, as well as matrix synthesis, cell proliferation and senescence markers in cell cultures derived from annulus fibrosus (AF) and nucleus pulposus (NP) isolated from the discs of young (6-9 months) and older (36-50 months) New Zealand White rabbits. Cellular bioenergetic parameters were measured using a Seahorse XFe96 Analyzer, in addition to quantitating mitochondrial morphological changes and membrane potential. Ageing reduced mitochondrial number and membrane potential in both cell types. Also, it significantly reduced glycolytic capacity, mitochondrial reserve capacity, maximum aerobic capacity and non-glucose-dependent respiration in NP. Moreover, NP cells exhibited age-related decline in matrix synthesis and reduced cellularity in older tissues. Despite a lack of changes in mitochondrial respiration with age, AF cells showed an increase in glycolysis and altered matrix production. While previous studies report age-related matrix degenerative changes in disc cells, the present study revealed, for the first time, that ageing affected mitochondrial number and function, particularly in NP cells. Consequently, age-related bioenergetic changes may contribute to the functional alterations in aged NP cells that underlie disc degeneration.

## Introduction

Increased life expectancy is rapidly expanding the proportion of aged individuals. Nearly a billion individuals (~ 11 % of the world population) are over the age of 65 years and this percentage is expected to double by 2050 (Chatterji et al., 2015). In high-income nations, disability in the ageing population amounts to nearly 50 % of the overall healthcare cost (Prince et al., 2015). Ageing of the musculoskeletal system is a leading contributor to the disease burden in the elderly population (Prince et al., 2015). In particular, lower back pain associated with age-related spinal degeneration is a leading cause of disability (Aigner et al., 2004; Cheung *et al.*, 2009) and imposes an annual economic burden of ~ 100 billion dollars in the United States alone (Dagenais et al., 2008).

Intervertebral disc degeneration is a common underlying cause of chronic disability and debilitating lower back pain in older adults. In the spine, age-related degeneration of the intervertebral disc occurs earlier than in other tissues (Vo et al., 2016). The intervertebral disc, a large fibrocartilage composite structure, plays a primary role in supporting spinal loading and facilitating multi-directional movement of the trunk and neck (Adams et al., 2009). The disc comprises two distinct regions with resident cells of different dermal lineages subjected to different nutritional and mechanical environments. The nucleus pulposus (NP) consists of cells at a relatively low density within a gelatinous, proteoglycan-rich matrix that swells under compressive loading to resist compression and dissipate applied loading (Roughley et al., 2002). The annulus fibrosus (AF) maintains a population of fibrochondrocytes within a fibrocartilaginous matrix organised in concentric lamellae of collagen fibres that constrain the swelling of the NP and anchor each disc to its adjacent vertebral bodies (Roughley, 2004).

The disc is a predominantly avascular structure that receives nutrition and exchanges metabolic waste by diffusion from capillaries in the subchondral bone (Huang et al., 2014). Age-related disc degeneration begins in the NP with a loss of its distinctive matrix components, principally proteoglycans (PGs). With increasing age, extracellular PGs become fragmented and lost from the disc, resulting in progressive disc dehydration (Roughley et al., 2002). This decreases the NP swelling pressure and load distribution within the disc, thereby exacerbating fibrosis of the NP and provoking subsequent remodelling of the surrounding AF (Adams et al., 2006). Age-related changes in vascular nutrient supply or diffusive transport, either in the disc, endplate or bone, may reduce both the transport of nutrients into the disc and disposal of acidic metabolites out of the disc, causing nutritional stress. Loss and phenotypic changes of disc cells drive degenerative changes in matrix proteoglycan composition and structure, which compromise the disc function (Singh et al., 2009).

Ageing is a molecular process whereby cells and extracellular matrix accumulate molecular damage over time and exhibit destructive and/or dysregulated responses to this accumulated damage, ultimately leading to impairment of normal physiology (Roughley et al., 2002; Vo et al., 2016). Molecular hallmarks of ageing are many but include mitochondrial dysfunction, increased senescence and impaired metabolism related to nutrient sensitivity (Lopez-Otin et al., 2013). Several notable factors are implicated as drivers of the intervertebral disc ageing. Oxidative damage is a mechanism involved in age-related disc degeneration (Nasto et al., 2013a). Associated inflammatory signalling, which is also elevated systemically in ageing, contributes to degeneration (Podichetty, 2007). Nutrient alteration or deprivation, suspected to be present in the ageing disc (Huang et al., 2014), drives cell phenotypic changes *in vitro* (Bibby et al., 2005) and is thought to contribute to disc degeneration *in vivo,* possibly through modulation of mTOR (Ito et al., 2017). Mitochondrial function can be influenced by factors associated with ageing, including oxidative damage, inflammatory stress and nutrient deprivation (Correia-Melo et al., 2016). Thus, the resultant disc degeneration may involve mitochondrial dysfunction (Gruber et al., 2013), which can negatively impact cellular processes, *e.g.* matrix synthesis and metabolic pathways. Hence, cellular bioenergetics analysis, which quantitatively assesses mitochondrial oxidative phosphorylation (OXPHOS) and glycolysis, will provide insights into the role of cellular metabolic decline in age-related disc degeneration.

Analysis of cellular bioenergetics provides a sensitive measure of metabolic changes related to phenotypic and pathologic changes in cells (Ho et al., 2012; Maynard et al., 2015). Although mitochondrial-derived reactive oxygen species (ROS) play a causal role in driving age-related intervertebral disc degeneration (Nasto et al., 2013a), it remains largely unknown how the aforementioned age-related changes in the disc environment (oxidative stress, inflammation, nutrient deprivation and senescence) impact and alter bioenergetics and mitochondrial function. While investigations of energy metabolism are performed in disc cells (Agrawal et al., 2007; Gruber et al., 2013), the baseline bioenergetic profiles of young, healthy AF and NP cells are still unknown. Moreover, little is known about disc cell mitochondria. In the present study, these questions were addressed by testing the hypothesis that ageing increased disc mitochondrial dysfunction, resulting in altered bioenergetics, which could negatively impact disc matrix homeostasis.

## Materials and Methods

### Cell isolation

Thoracolumbar spines were extracted from young (6-9 months) and older (36-50 months) female New Zealand White rabbits (Covance, Denver, PA, USA) following euthanasia. The study was approved by the Institutional Animal Care and Use Committee of the University of Pittsburgh (14073827). For all cellular experiments, intervertebral discs were isolated from spines and the AF and NP tissues were manually separated. Disc tissues were separately subjected to 1 h digestion with 0.2 % pronase (Calbiochem) in F-12 medium supplemented with 5 % foetal bovine serum (FBS) and 1 % penicillin/streptomycin (P/S) followed by overnight digestion at 37 °C in 0.02 % Collagenase P (Roche) in F-12 medium supplemented with 5 % FBS and 1 % P/S. Then, cells were isolated from remaining tissue and debris through a 70 μm strainer, pelleted, re-suspended in growth medium (F-12 with 10 % FBS and 1 % P/S), plated onto T-75 flasks and grown to 90 % confluence. Next, cells were detached from the plates by 0.05 % trypsinethylenediaminetetraacetic acid (EDTA) (Gibco), counted and plated for subsequent experiments at passage 1.

### Matrix protein synthesis by radiolabel incorporation

As described previously (Wang et al., 2012), cells were cultured for 3 d in F-12 medium containing 10 % FBS and 1 % P/S in triplicate in a 48-well plate. 20 μCi/mL of ^35^S-sulphate and 10 μCi/mL ^3^H-L-proline were added to the cell medium to quantify PG and collagen synthesis, respectively. Afterward, cells were homogenised for 1 h at 4 °C by shaking them in 200 mM sodium chloride, 50 mM sodium acetate, 0.1 % Triton X-100 (X-100, Sigma-Aldrich), 10 mM EDTA, 50 μm dithiothreitol (DTT) (D9779, Sigma-Aldrich) and 1× protease inhibitor (P8340, Sigma-Aldrich). Then, PGs were extracted from the homogenate by addition of an extraction buffer containing 8 M guanidine hydrochloride (G3272, Sigma-Aldrich), 50 mM sodium acetate, 10 mM EDTA and 1× protease inhibitor and vigorous shaking for 4 h at 4 °C. Insoluble protein containing collagen remained in the homogenate. For PG synthesis, samples were mixed for 1 h at room temperature with alcian blue (0.02 %), loaded onto a nitrocellulose membrane and washed to remove unincorporated radiolabel. For collagen synthesis, the collagenase-sensitivity assay was performed as previously described (Wang et al., 2012). Samples were loaded within a scintillation counter (Tri-Carb 2100TR, PerkinElmer) where counts per minute (CPM) were measured and converted to the number of pmol of sulphate or L-proline. Results were normalised to DNA amount as measured using the Quant-iT^™^ PicoGreen^™^ dsDNA Assay Kit (P7589, Life Technologies).

### Cell proliferation by Cell Counting Kit-8 (CCK-8) assay

Disc cell proliferation was measured using the CCK-8 assay (CK04, Dojindo Molecular Technologies, Inc., Rockville, MD, USA), following the manufacturer’s instructions. Briefly, AF and NP passage 1 cells were plated at 1 × 10^3^ cells per well in a 96-well plate in triplicate. Every 24 h after plating (0-10 d), 100 μL of fresh F-12 medium (supplemented with 10 % FBS and 1 % P/S) plus 10 μL of CCK-8 solution were added to the cells and absorbance was measured at 450 nm using a plate spectrophotometer (PerkinElmer).

### Bioenergetic flux measurement by Seahorse XFe96 Analyzer

Methods developed for bioenergetics measurements of adherent cells using a Seahorse Extracellular Flux Analyzer have been adapted to disc cells (de Moura and Van Houten, 2014; Qian and Van Houten, 2010). Passage 1 AF and NP cells were plated at 10-15 × 10^3^ cells per well on XFe96 plates and cultured for 3 d in F-12 medium (10 % FBS and 1 % P/S). Just prior to analysis in a Seahorse Analyzer XFe96 (Agilent Technologies), cell medium was changed to unbuffered Dulbecco’s modified Eagle’s medium (DMEM; Sigma-Aldrich) supplemented with 2 mM Glutamax-1 (Gibco), 1 mM sodium pyruvate (Sigma-Aldrich), 25 mM glucose (Sigma-Aldrich), 32 mM sodium chloride (Sigma-Aldrich) and 15 mg phenol red (Sigma-Aldrich). Extracellular flux measurements were acquired three to four times at 6 min intervals over five different treatment conditions: basal, 1 μM oligomycin, 300 nM carbonyl cyanide-4-(trifluoromethoxy) phenylhydrazone (FCCP), 100 mM 2-deoxglucose (2DG) and 1 μM rotenone. Oxygen consumption rate (OCR) and extracellular acidification rate (ECAR) were calculated at each measurement and normalised to protein amount per well using the Crystal Violet dye (C3886, Sigma-Aldrich). Glycolytic capacity was calculated by subtracting the basal ECAR value from the ECAR value after oligomycin treatment. Individual parameters of OXPHOS were derived from OCR profiles (see Fig. 4a,b) as described by de Moura and Van Houten (2014) and shown in Table 1.

### Immunofluorescent staining

Cells were briefly washed twice with cold phosphate-buffered saline (PBS) and fixed in 2 % paraformaldehyde in PBS for 15 min at room temperature. Next, they were blocked and semi-permeabilised with 0.25 % Triton X-100, 20 % serum (from the species in which the secondary antibodies were made) and 1 % bovine serum albumin (BSA) in PBS for 30 min at room temperature. Then, cells were incubated with the specific primary antibodies for lamin-B1 (AB16048; Abcam), H2A histone family member X (H2AX) (05-636; Millipore) and ATP synthase β (MAI-930; ThermoFischer Scientific) overnight at 4 °C. Subsequently, they were washed three times with PBS and incubated in Donkey anti-Rabbit IgG (H+L) Highly Cross-Adsorbed secondary antibody, Alexa Fluor^®^ 488 (A21206; Life Technologies) for 30 min at room temperature, according to the manufacturer’s protocol, and visualised using an inverted fluorescent microscope (Eclipse TE2000-U, Nikon Inc.). Cells were counter-stained with DAPI to obtain the number of viable cells and the percentage of cells positive for lamin-B1 was calculated.

### Senescence-associated β-galactosidase staining (SA-β-gal)

SA-β-gal staining was performed as previously described (Dimri et al., 1995). Images were acquired by brightfield microscopy at 10× magnification. The percentage of SA-β-gal-positive cells was calculated by dividing the number of SA-β-gal-positive cells by the total number of cells and multiplying by 100.

### Mitochondrial quantification

To assess mitochondrial morphology, immunofluorescent analysis of fixed cells was performed using antibodies against translocase of the outer membrane 20 (TOM20; SC-11416; Santa Cruz) or ATP synthase (MA1-930; ThermoFisher Scientific). Cells were co-stained with phalloidin-cy3 (actin, Life Technologies, R415) and DAPI (nuclei). Confocal z-stacks were collected using a 60× (1.43NA) objective on a Nikon A1 equipped with GASP detectors and NIS Elements software (Nikon Inc.). The confocal datasets were imported into Imaris (Bitplane, Zurich, Switzerland) for surface rendering and calculation of mitochondrial volumes.

### Mitochondrial potential

Cells were seeded on 35 mm glass-bottom dishes (MatTek Corporation, Ashland, MA, USA) and incubated with 10 mg/mL mitochondrial membrane potential probe (JC-1; ThermoFisher Scientific) for 15 min at 37 °C. Cells were washed with PBS, the medium replaced and the dish inserted into a closed, thermo-controlled (37 °C) stage-top incubator (Tokai Hit Co., Shizuoka-ken, Japan) on top of the motorised stage of an inverted Nikon TiE fluorescent microscope equipped with a 60× oil immersion objective (CFI PlanFluor, NA 1.43; Nikon Inc.) and NIS Elements software (Nikon Inc.). Dual wavelength excitation was achieved using a Lumencor diode-pumped light engine (SpectraX, Lumencor Inc., Beaverton, OR, USA) and detected using an ORCA-Flash 4.0 sCMOS camera (HAMAMATSU Corporation, Bridgewater, NJ, USA) and excitation and emission filters from Chroma Technology Corporation (Bellows Falls, VT, USA). Data were collected from approximately 5 to 10 cells per stage position, with 15-20 stage positions in each of the separate experiments. Data were analysed using NIS Elements software (Nikon Inc.).

### Statistical analysis

Wilcoxon rank-sum test was used to compare samples from young and older donors for GAG, matrix synthesis and bioenergetics outcomes with statistical significance set at *p* < 0.05. Cell proliferation data from young and older donors were compared by performing a linear regression modelling of the curves’ linear growth phase and applying an analysis of the variance to the linear models to assess whether the models were distinct. Statistical analyses were performed in R 3.2.1 (R Foundation for Statistical Computing). All data were presented as mean ± standard error of the mean (SEM).

## Results

### Aged discs displayed decreased cellularity and increased senescence

**Fig. 1.**
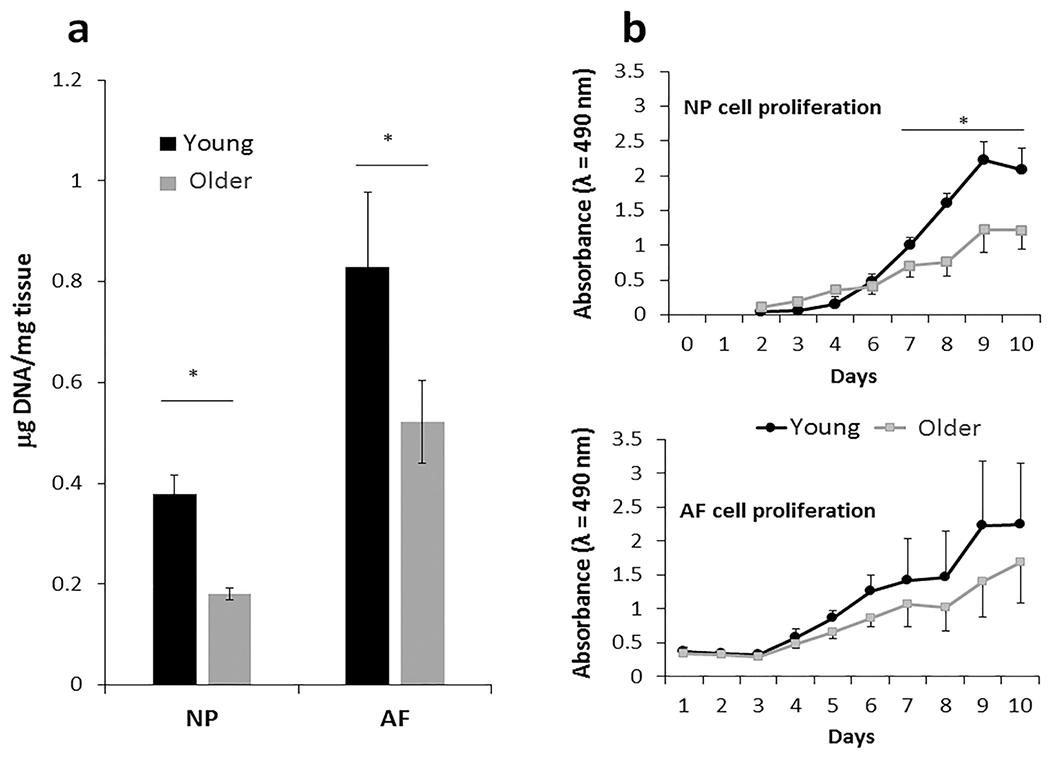
Decreased cellularity and cell proliferation in aged disc tissue. (**a**) Cellularity, measured as amount of DNA per disc tissue mass, was less in NP and AF of young (6-9 months) than older (> 3 year-old) rabbits. Error bars represent SEM. (**b**) Older nucleus NP (days 7-10) and AF (days 5-10) cells in their linear growth region exhibited statistically significant lower proliferative capacity than young cells, based on a linear regression model; *n* = 4 samples, * *p* < 0.05.

New Zealand White rabbits were used since they are a commonly used model of human intervertebral disc degeneration and provide sufficient quantities of disc cells for characterisation (Leckie et al., 2013; Sowa et al., 2012). Young (6-9 months) and older (36-50 months) female rabbits, which have an average lifespan of about five years, were used. Cellularity, measured by the amount of DNA per tissue mass, was reduced in both the NP and AF tissues of aged discs as compared to young discs (Fig. 1a). Functionally, this might impact the disc health since each cell had to sustain a larger region of surrounding matrix.

### Reduced cellularity, as observed in the older rabbits, might reflect cell death or loss of proliferation

**Fig. 2.**
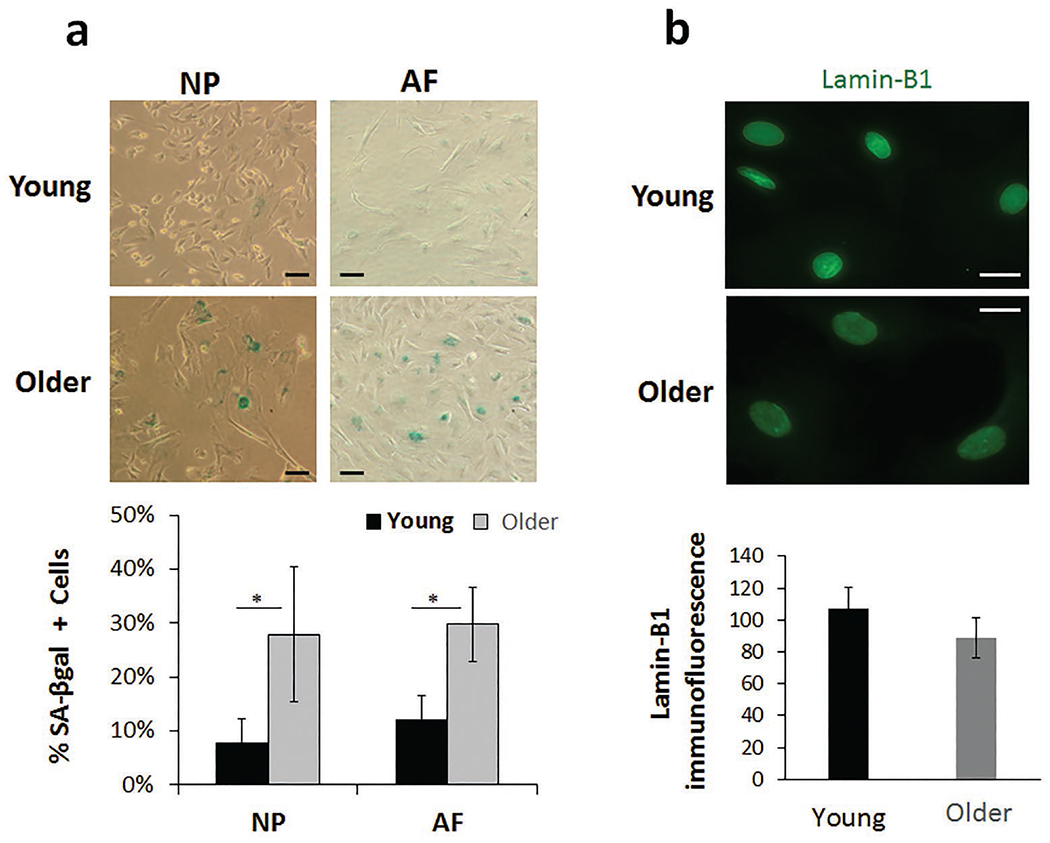
Increased expression of markers of cellular senescence in aged disc cells. (**a**) Representative images of SA-β-Gal staining (top) and quantified percentage of positive (blue) cells for SA-β-Gal activity (bottom) in young and older disc cells. (**b**) Reduced immunofluorescence of lamin-B1 (green), a marker of cellular senescence, was observed in older NP cells as compared to young NP cells. Results are expressed as mean of four different samples ± SEM, * *p* < 0.05, scale bars: 50 μm.

Reduced proliferation, as determined by CCK-8 cell proliferation assay, was observed in primary disc cell culture derived from the older rabbits, particularly from NP cells (Fig. 1b). Decreased proliferation of older disc cells could be due to an increased proportion of senescent cells, which are cell cycle arrested. The percentage of senescent disc cells was assessed by senescence-associated β galactosidase (SA-β-Gal) staining of passage 1 NP and AF cells. About 10 % of the NP and AF cells stained positively for SA-β-Gal in the young rabbits, whereas this number increased 3-fold in the older rabbits (Fig. 2). Decreased lamin-B1, another marker of cellular senescence, was observed in older NP cells (Fig 2b). Disc cells *in situ* are largely non-proliferative, but they become proliferative at the site of injury to aid repair and regeneration (Johnson et al., 2001;Roberts et al., 2006). The observations of the present study were consistent with disc cell proliferative capacity decreasing with age due, at least in part, to elevated disc cellular senescence (Gruber et al., 2006). The study’s findings were also consistent with existing evidence of higher incidence of cellular senescence in degenerative human discs and discs in murine model of accelerated ageing (Le Maitre et al., 2007; Nasto et al., 2013b).

### Aged discs displayed altered matrix production

**Fig. 3.**
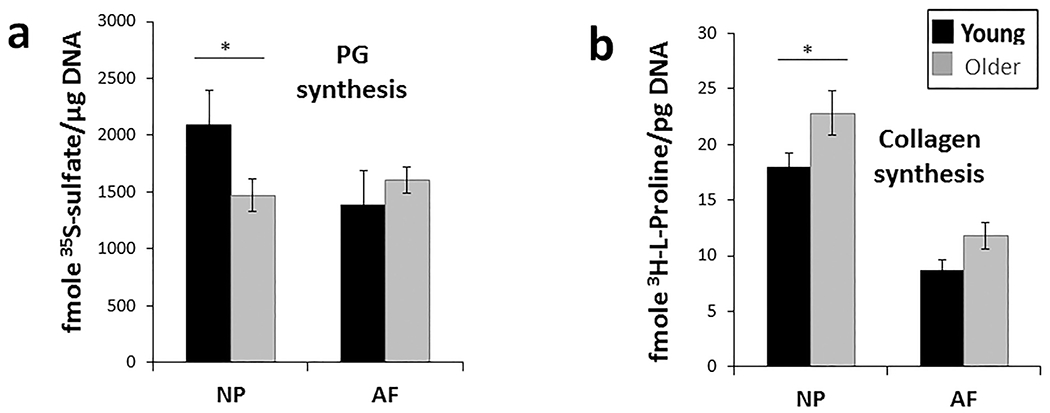
Matrix synthesis, a measure of energy demand, changed with age. Older (> 3 year-old) NP cells had lower PG synthesis rate (radio-labelled ^35^S-sulphate incorporation) but higher collagen synthesis rate (radio-labelled ^3^H-proline incorporation) than young (6-9 months) cells, while AF cells showed no significant age-related changes in synthesis. Results are expressed as mean of four different samples ± SEM, * *p* < 0.05.

A major function of healthy disc cells is to synthesise extracellular matrix (Gruber et al., 2004; Naqvi SM and Buckley CT, 2004; Potier et al., 2014). To further examine cellular changes with age, the capability of disc cells from young and older rabbits to produce matrix proteins was measured. Disc cells were isolated and ^35^S-sulphate and ^3^H-L-proline radiolabelling assays performed to quantify the new active synthesis of the disc primary matrix macromolecules, PGs and collagen. Compared to young NP cells, older NP cells showed a 25 % decline in PG synthesis and a 21 % increase in collagen synthesis (Fig. 3). A decrease in NP cell PG synthesis matched the expectations based on an early decline in NP PG content in age-related disc degeneration (Roughley et al., 2002). Similarly, elevated collagen synthesis in NP cells reflected a more fibrotic phenotype associated with ageing and degeneration (Kaapa et al., 1995). Older AF cells exhibited no significant changes in their capacity to synthesise collagen or PGs (Fig. 3).

### Aged disc cells displayed altered cellular bioenergetic profiles

**Fig. 4.**
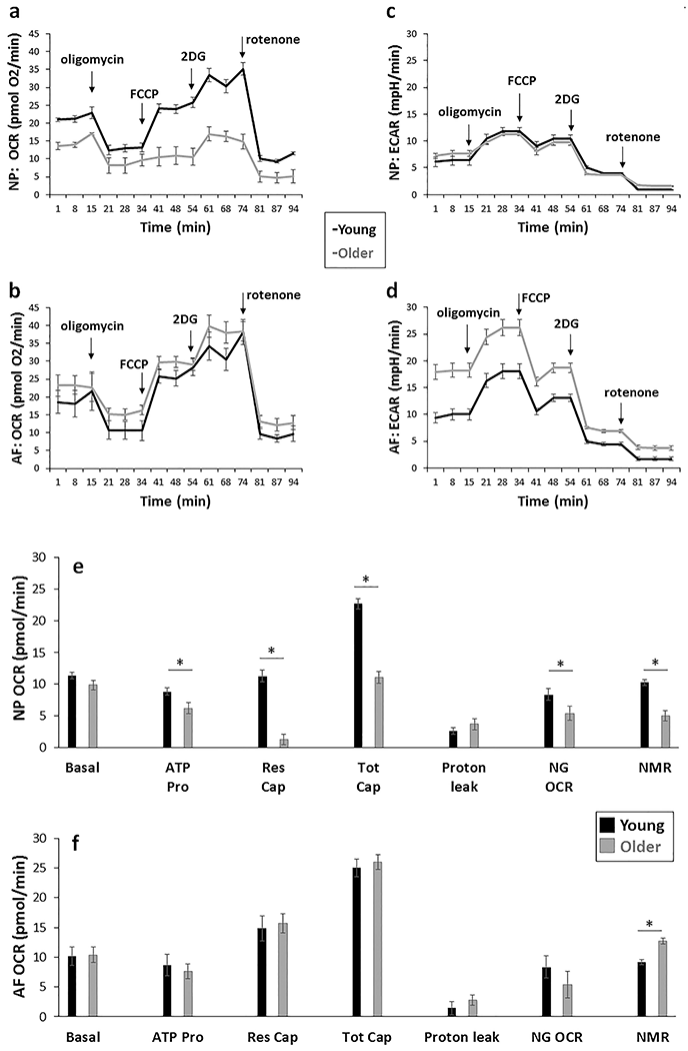
Pharmacological profiling of OCR and ECAR of young and older disc cells. OCR and ECAR of NP (**a,c,** respectively) and AF cells (**b,d,** respectively) were measured by Seahorse XFe96 Extracellular Flux Analyzer at basal conditions and with serial administration of 1 μM oligomycin, 0.3 μM FCCP, 100 mM 2DG and 1 μM rotenone. Results are expressed as a mean of four different samples ± SEM. Individual parameters of OXPHOS were derived from OCR profiles of young and older (**e**) NP and (**f**) AF cells, as described in Materials and Methods. Older NP cells, but not older AF cells, showed declines in mitochondrion-dependent ATP production (ATP Pro), respiration reserved capacity (Res Cap), respiration total capacity (Tot Cap), non-glucose respiration (NG OCR) and non-mitochondrial oxygen consumption (NMR). Results are expressed as mean of four different samples (derived from four rabbits) ± SEM, * *p* < 0.05.

**Fig. 5.**
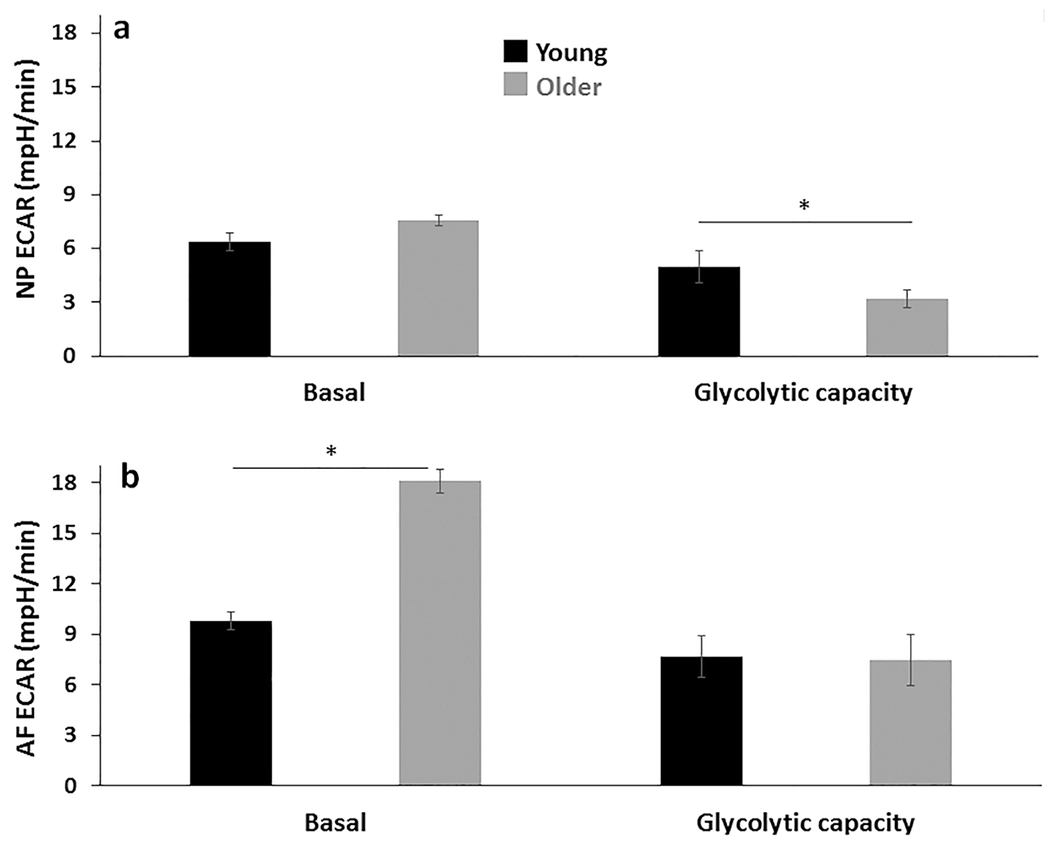
Ageing caused profound changes in glycolysis in both NP an AF cells. NP cells showed reduced glycolytic capacity. Basal and glycolytic capacity were derived from ECAR profiles of young and older AF and NP cells. Reduced glycolytic capacity (the increase in glycolysis after inhibiting OXPHOS with oligomycin) was observed in older NP cells as compared to young ones, while basal ECAR was significantly elevated in older AF cells as compared to young AF cells. Results are expressed as mean of four different samples ± SEM, * *p* < 0.05.

To determine if the bioenergetic profiles were altered in disc cells with ageing, specific features of OXPHOS and glycolysis in AF and NP cells from young and older rabbits were measured using a Seahorse XFe96 Extracellular Flux Analyzer (Fig. 4). OCR, which reflects the level of OXPHOS (Qian et al., 2010), and ECAR, which is used as a measure of glycolysis (Qian et al., 2010), were measured at basal conditions and following addition of specific inhibitors of the electron transport chain (see Bioenergetic flux measurement by Seahorse XFe96 Analyzer). OCR but not ECAR profile in NP cells showed a significant decline with age (Fig. 4a,c). In contrast, AF cells showed no significant changes in OCR, but a 2-fold increase in basal levels of glycolysis with ageing (Fig. 4b,d and Fig. 5b).

NP cells from older rabbits displayed a 5-fold decline in reserve capacity and a 2-fold decrease in maximum respiratory capacity, while other individual parameters of OXPHOS, including ATP production, non-glucose respiration, non-mitochondrial oxygen consumption, also decreased significantly with ageing in NP cells by 31 ± 6 %, 40 ± 14 % and 52 ± 17 %, respectively (Fig. 4e). Moreover, aged NP cells exhibited a modest decline in glycolytic capacity (Fig. 5). Except for lowered non-mitochondrial oxygen consumption, AF cells from older rabbits showed no decline in OXPHOS but a significantly higher basal ECAR level (2-fold) than AF cells from young rabbits (Fig. 5). These findings suggested that OXPHOS was affected by ageing in NP cells to a greater extent than in AF cells.

### Aged disc cells exhibited altered mitochondrial abundance, morphology and function

**Fig. 6.**
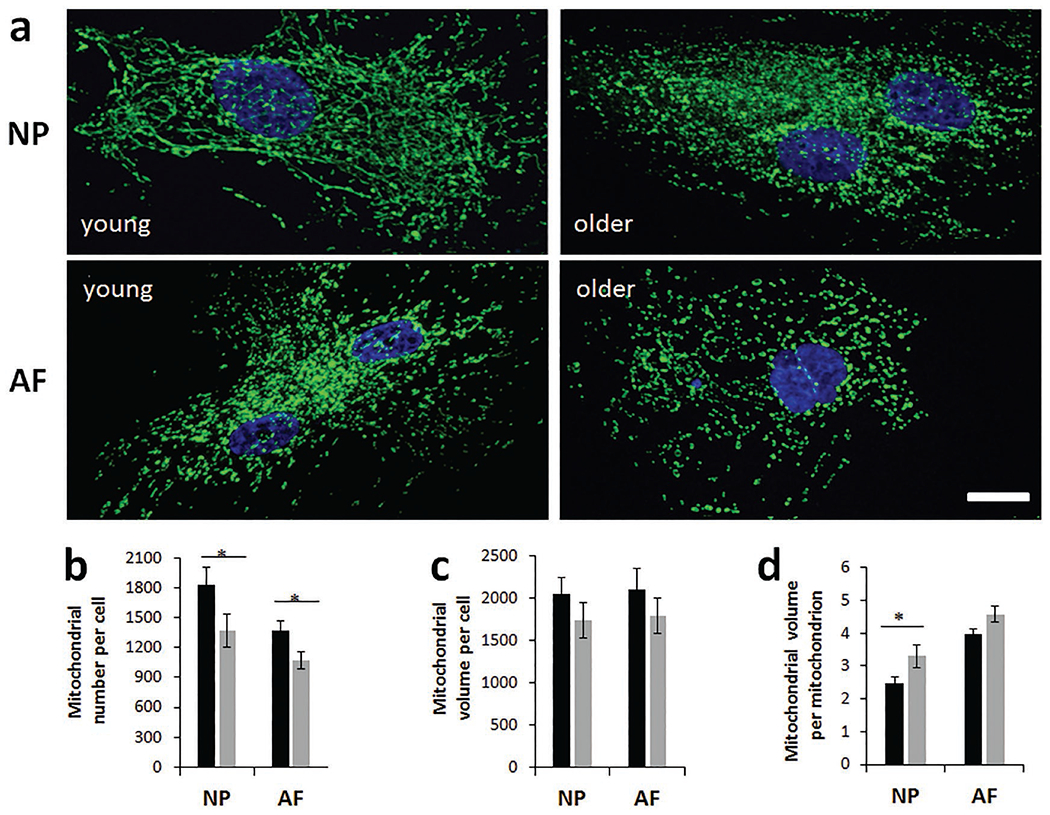
Mitochondrial confocal immunofluorescent imaging illustrated cell and age-dependent changes in mitochondrial count and volume. (**a**) Confocal z-stacks with ATP-synthase-labelled mitochondria and DAPI-stained nuclei for young and older NP and AF cells. (**b**) Mean quantified mitochondrial count per cell, (**c**) mean overall mitochondrial volume per cell and (**d**) mean mitochondrial volume per mitochondrion are graphically represented. Results are expressed as mean of three different experiments ± SEM, * *p* < 0.05, scale bars: 10 μm.

The decline in OXPHOS parameters in NP cells might arise from loss of mitochondria or mitochondrial function. Mitochondrial number and volume were measured in disc cells by confocal-microscopy-reconstructed z-stacks (Fig. 6). Mitochondrial number per cell decreased in aged NP cells (25 ± 12 %) and AF cells (22 ± 9 %) as compared to cells from young animals (Fig. 6b). Mitochondrial volume per cell also trended toward a decline in both NP (15±3 %) and AF (15 ± 6 %) aged cells (Fig. 6c). Mean volume per mitochondrion, a measurement of mitochondrial fragmentation or fission [which is associated with mitochondrial dysfunction (Qian and Van Houten, 2010)], increased significantly in aged NP cells by 33 ± 18 % (Fig. 6d).

**Fig. 7.**
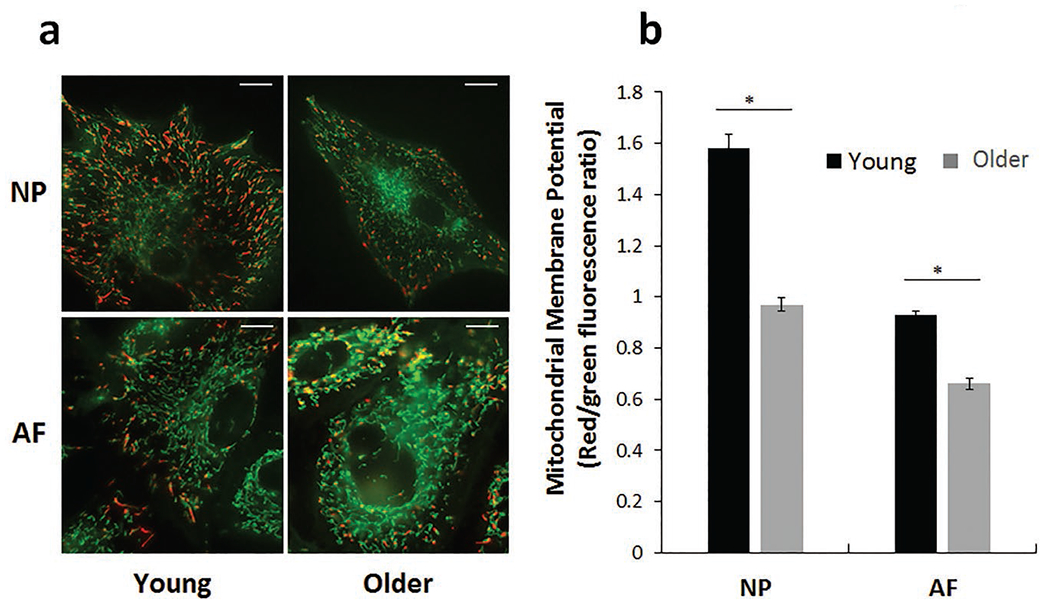
Mitochondrial membrane potential, quantified by JC-1 staining, pointed to a decrease in membrane potential with age. (**a**) Representative young and older NP and AF cells, (**b**) with averaged results across multiple fields-of-view within a single culture for AF and NP cells. Results are expressed as mean of three different experiments ± SEM, * *p* < 0.05, scale bars: 10 μm.

The most striking age-related mitochondrial change in disc cells was the mitochondrial membrane potential. Mitochondrial membrane potential, an indicator of mitochondrial health and function (Sakamuru et al., 2016), decreased significantly by 39 ± 7 % and 29 ± 8 % in NP and AF cells, respectively (Fig. 7). In summary, older NP cells showed evidence of mitochondrial loss and dysfunction, which might contribute to the observed bioenergetic decline in aged NP cells. In aged AF cells, mitochondrial changes were milder and OXPHOS not affected.

## Discussion

The present study examined how ageing altered disc cell function, mitochondria and bioenergetic profiles. Disc cells play a crucial role in the preservation of intervertebral disc integrity and function by maintaining the extracellular matrix through continued synthesis of matrix proteins (Bayliss et al., 1998; Johnstone et al., 1993; Johnstone et al., 1995). This imposes significant energy demands on disc cells. With ageing, expression of anabolic and matrix synthesis genes declines (Singh et al., 2009). The present study examined whether there was an age-related functional change in mitochondria and bioenergetic profiles in disc cells. For the first time, mitochondrial number and bioenergetic profiles were demonstrated to decline with age, particularly in NP cells, and to correlate with a loss of disc cells and their function. Age-related disc cell mitochondrial and bioenergetic changes might contribute to the loss of matrix homeostasis that underlay disc degeneration. However, it is important to note that the cell culture results should be confirmed *in vivo*.

Glycolysis and OXPHOS represent two major bioenergetic and metabolic processes that cells use to generate ATP and important metabolic precursors (Mitchell, 1961; Romano and Conway, 1996). The assessment of these two processes using the Seahorse technology (de Moura and Van Houten, 2014) revealed a significant decline in OXPHOS in NP cells isolated from older rabbits. In contrast, AF cells from older animals showed altered mitochondrial morphology and lower membrane potential but no change in mitochondrial respiration. However, AF cells did show a profound increase in glycolysis. In contrast, in NP cells from older rabbits, there was a profound decrease in OXPHOS, without a concomitant increase in glycolysis. NP cells from older rabbits exhibited a significant decline in bioenergetic reserve and maximum respiratory capacity. Bioenergetic reserve capacity is a valid predictor of how cells will respond to stress, *e.g.* oxidative stress (Hill et al., 2009). Loss of reserve capacity may lead to protein damage and cell death (Dranka et al., 2010; Hill et al., 2009). The decline in reserve and maximum capacity with ageing likely impacted the ability of NP cells to respond adequately to environmental stresses, such as injury and inflammation. These limited mitochondrial capacities could hamper matrix repair or cellular stress responses.

2DG caused an increase in OXPHOS, which might represent the use of an alternative carbon source, such as glutamine or fatty acid beta-oxidation, for energy supply in the mitochondrion. Ovarian tumour cells show an increased demand for fatty acids and fatty-acid beta-oxidation (Nieman et al., 2013). Similarly, the decline in non-glucose OCR in older cells represented a loss of metabolic flexibility, whereby younger NP cells can activate metabolic processes for oxidation of alternative carbon sources (Agrawal et al., 2007). This loss of metabolic flexibility could impair responses to nutritional stresses likely to occur in the disc with ageing. The lack of change in basal OCR emphasised the important principle of stressing a system to measure its functional reserve. The observation that NP cells were affected more dramatically than AF cells supported the concept that the progression of age-associated degenerative changes occurs first in the NP and, then, spreads to the AF (Roughley et al., 2002). It should be noted that both young AF and NP cells exhibited considerable degree of non-mitochondrial oxygen consumption. It is unclear how disc cells use non-mitochondrial oxygen, but non-mitochondrial oxygen usage may reflect free radicals generated by other oxidases, such as nicotinamide adenine dinucleotide phosphate (NADPH) oxidase or xanthine oxidase (Banh et al., 2016).

Changes in bioenergetic parameters reflect alterations in mitochondrial function (Madeira, 2012). Hence, the mitochondria were investigated searching for evidence of structural damage or dysfunction associated with age. The role of the mitochondria in intervertebral disc cells has been understudied but given their role role in ageing, it has recently become a more active area of research in disc ageing (Gruber et al., 2013; Nasto et al., 2013a; Shen et al., 2017). The present study supported i) the role of the mitochondria in disc cellular respiration and ii) a decline of mitochondrial function in ageing discs. In NP, mitochondria are relatively low in abundance (Gan et al., 2003) but functional (Agrawal et al., 2007) and they do contribute significantly to the energy supply in the presence of glucose (Gruber et al., 2015; Ishihar and Urban, 1999). Hypoxia-inducible factor (HIF)-1α regulates reducing agents [*e.g.* reduced nicotinamide adenine dinucleotide (NADH)] entering the mitochondrion as well as the expression of the electron transport chain enzyme cytochrome c oxidase-4, thereby suppressing the OXPHOS (Risbud et al., 2010). In addition to producing ATP, mitochondria are essential for other cellular processes, including synthesis of important metabolites (*e.g.* nucleotides, lipids, Fe-S centres for redox reactions), calcium homeostasis, mitophagy and apoptosis (Dorn and Kitsis, 2015). The present study showed that rabbit NP cells utilised mitochondria for energy supply *in vitro,* even under conditions of low oxygen. Also, NP cells from aged rabbits had an impaired capacity to respond to metabolic stress. This correlated with an impaired ability to proliferate and produce matrix. The trend toward decreased mitochondrial number and lower mitochondrial membrane potential in NP cells from older rabbits suggested that mitochondrial dysfunction might explain some of the bioenergetic changes observed in aged disc cells. This lower membrane potential was consistent with increased proton flux across the mitochondrial membrane. These mitochondrial changes in NP cells could contribute to the observed cellular changes associated with age-related degeneration.

In human discs, mitochondria in AF cells are functional and their capacity for aerobic respiration declines in concert with the severity or grade of degeneration (Gruber et al., 2013). The study’s results confirmed that AF mitochondria played a prominent role in respiration. However, a non-age-related decline in OXPHOS in the rabbit model was observed. Gruber et al. (2013) report a decline in mitochondrial mass and increased mitochondrial damage (branching, small cristae, poorly defined cristae, swollen cristae within dark mitochondria) in degenerated AF samples. Only a modest decline in mitochondrial number and membrane potential was observed in AF cells from older rabbits. Nonetheless, these changes reflected the observations of damaged, inefficient mitochondria in aged rabbit AF samples. Those observations were consistent with the notion that NP defects precede AF (Adam and Roughley, 2006) and that only early changes in age-related AF mitochondria and bioenergetic changes were observed in the present study.

The present study examined ageing in both resident cell populations, AF and NP, of the intervertebral disc, an organ that ages quite rapidly in humans (Boos et al., 2002; Miller et al., 1988; Tang et al., 2016). The observed changes in NP cells (reduced matrix synthesis, decreased proliferative capacity and elevated SA-β-Gal activity) reflected age-related degenerative changes. This typically begins in the NP with PG loss, resulting in dehydration and increased fibrotic matrix deposition (Roughley et al., 2006). These changes elicit degenerative cellular changes, including increased fibrosis and cell death (Iatridis et al., 2006). Reduced PG synthesis and increased collagen synthesis were observed in older NP cells, the latter driving the fibrotic phenotype. This increased collagen and fibrosis indicated a maladaptive response to injury that is observed broadly in ageing tissues (Kurundkar and Thannickal, 2016; Parola and Robino, 2001). The current study’s findings were consistent with previous studies demonstrating that PG content in disc declines with age (Sivan et al., 2014) while collagen content increases (Olczyk, 1992). Indeed, increased collagen synthesis in NP cells is observed in degeneration models (Kaapa et al., 1995) and progeroid mice exhibit lower proteoglycan synthesis (Nasto et al., 2012). It is possible that impaired mitochondrial function and altered bioenergetics observed in the present study might contribute to age-related alterations in disc matrix production. This was consistent with previous studies reporting that lower energy reserves (*i.e.* ATP) in cells cause reduced proteoglycan synthesis (Kanwar et al., 1992; Mason and Sweeney, 1994).

An increase in the fraction of senescent cells was observed in populations isolated from aged rabbits NP and AF relative to young animals. This will clearly affect the proliferative capacity of cells in the disc, as observed. However, the senescent cells might also impact other aspects of disc cell physiology through their secretory phenotype. This is an interesting area of research that should be pursued. The study’s findings were consistent with a previous model of the relationship between senescence in disc cells and ageing (Wang et al., 2016) and a study from Gruber et al. (2001) reporting a significant but relatively small role of age on the proliferative capacity of disc cell.

## Conclusions

The study’s findings demonstrated that ageing altered the bioenergetic profiles of intervertebral disc cells, particularly NP cells. Also, ageing reduced mitochondrial number, volume and function, supporting a model in which modest mitochondrial dysfunction underlaid bioenergetic changes, which in turn affected cellular function. Changes in matrix synthesis, cellularity and proliferative capacity correlated with the decline in NP cells’ bioenergetic profiles. Future studies are needed to establish whether mitochondrial changes, bioenergetics changes or cellular senescence are the initiator of age-related degenerative disc disease.

## Figures and Tables

**Table 1.** Parameters of OXPHOS derived from the oxygen consumption rate profile obtained by Seahorse Extracellular Flux Analyzer.

Parameters	Abbreviation	Calculation
Mitochondria-dependent ATP production	ATP Pro	OCR after oligomycin addition - basal OCR
Reserved capacity	Res Cap	OCR after 2DG addition – basal OCR
Respiration total capacity	Tot Cap	OCR after 2DG addition – OCR after rotenone addition
Proton leak	Proton Leak	OCR after oligomycin addition – OCR after rotenone addition
Non-glucose respiration	NG OCR	OCR after 2DG addition – OCR after FCCP addition
Non-mitochondrial oxygen consumption	NMR	OCR after rotenone addition

## Discussion with Reviewer

Dimitris Kletsas Do the changes in bioenergetics during ageing of the intervertebral disc presented here reflected similar changes in others tissues?

Authors Yes and no, depending on the tissue types.

Dimitris Kletsas As similar changes in mitochondria are observed in both NP and AF cells, how did this lead to totally different changes in bioenergetic profiles?

Authors While AF and NP cells showed similar age-related changes in the mitochondrion, the changes were more pronounced in NP cells. For example, aged NP cells showed increased mitochondrial fragmentation (Fig. 6d) and larger reduction in mitochondrial number (Fig. 6a) and mitochondrial membrane potential (Fig. 7) than AF cells. This could explain the reduction in OXPHOS in aged NP, but not AF, cells.
